# A Positive and Direct Method of Casting and Adapting Aluminum Plates to Fit

**Published:** 1918-05

**Authors:** G. P. Baughman


### A POSITIVE AND DIRECT METHOD OF CASTING AND ADAPTING ALUMINUM PLATES TO FIT

BY G. P. BAUGHMAN, D.D.S.

Aluminum appeals to me as being the nearest to an ideal metal for dentures, as it is light, tough, a good conductor of thermal changes and is not expensive, permitting it to be used in nearly every case.

After having tried several methods of casting aluminum plates without positive results, I combined what appeared to be the good parts of the different methods with a few of my own invention and succeeded in getting an outfit with which I seldom fail to get complete castings.

My casting outfit consists of an iron vulcanite flask with an iron crucible attached to the lid, a piece of iron pipe containing some mouldine, and a means of applying heat enought to heat the flask to the proper degree and to melt the aluminum.

Adapt ordinary base plate wax closely to the model following the curve along the rim and trim off flush with the model. Thin the base plate wax over the posterior part of the palate to permit of easy swaging while plate is being adapted, also thin wax on anterior part of ridge if process is full and teeth are to be set well back. Build flange around lingual side of ridge clear over tuberosities to upper edge of rim, being sure it forms an undercut for retention of the vulcanite.

Form rolls of sticky wax about the size of a knitting needle, hold end over flame long enough for small bead to form and before it drops lightly touch to rim one-sixteenth of an inch below curved edge of the wax base and it will adhere, forming a wax bead which will be reproduced on the cast base in aluminum and will form a strong attachment for the vulcanite. Place as many of these beads all along the rim as may be needed. This form of attachment is easy to make and is quite secure; but if more is desired turn up crescent shaped flanges between the beads and the inner flange or build undercut rim around upper edge. Saturate model with its wax base plate in place with water and invest in the lower half of the flask extending the investment over the edge of the wax plate. After it has hardened sufficiently, smooth investment so the upper half of the flask will fit the lower closely all around.

Attach a roll of wax for the sprues, about the size of a match, to each tuberosity and one to the anterior median line of ridge and one to center of hard palate, and all must be united at the center of the flask one-eighth or one-fourth inch below the lid and at the center of the crucible.

After sprues are fixed give the investment in lower half of flask a coat separating medium and pour in investment, being careful to have no air spaces, and then adjust the lid of flask.

After investment has hardened sufficiently heat the flask until the wax becomes soft enough to avoid breaking any of the flanges and rims in separating. After separating, scald out wax with boiling water, and take small twist drill or other suitable instrument and perforate the investment into the iron crucible through the hole made by the wax sprues and pour boiling water through to be sure it is open.

If the two edges of flask fit closely all around they can now be bolted firmly together but if they are not closely in contact all around, whitewash the lower edge with a watery solution of the investment before bolting together. Heat the case over stove or flame until no more moisture is indicated in the crucible. Place a Brophy yoke burner or a double Bunsen gas flame on a level with the flask and direct the heat against the crucible on two sides, and while it is heating cut strips of aluminum plate and roll up into bundles and drop into crucible until fused. Add enough aluminum to cause the crucible to be a little over one-half full of melted aluminum.

Have the "gas pipe plunger" containing the moldine ready and as soon as you have moved aside the yoke burner, place the pipe over the crucible and exert strong, steady pressure with both hands two or three minutes. The metal remains liquid for some time and the steady pressure forces it into every crevice, and the numerous sprues permit it to circulate freely, the investment being porous permits the air to escape and the consequence is that in nearly every case there is a complete casting. Cool moderately and remove from the flask, saw off sprues, grind or file off smooth, then scrape down with large round sharp smooth edged vulcanite scraper and polish with felt wheel and pumice as easily as you would finish a vulcanite plate. Can give it a frosted appearance with concentrated lye, but do it quickly and wash off every trace or it will affect the metal.

Any heat which will cause the aluminum to melt in crucible will be sufficient. Have always used Bunsen burner with small flame directed against bottom of flask, while forcing the metal into mould.

Molten aluminum is sluggish and I think a sharper casting is obtained by keeping investment hot while casting. The wax could be burned out but requires considerable heat to burn out sufficient to get a sharp cast and the smoke from wax is objectionable, so the scalding out with hot water is preferred.

After getting a casting it is found sometimes that the blank will not stick, although an impression had been obtained that was quite satisfactory. It may be that the detrimental change took place while the metal was cooling from contraction of the arch at the heel of the plate. Turning the edge up with pliers may remedy this, but a more accurate way is as follows: If it is found that the base is too long, trim it so it will extend just back of the line dividing the hard and soft palate; then attach a roll of soft beeswax about the size of a match over the arch just over the posterior edge; warm thoroughly and insert in the mouth and press up firmly. Do this twice and in nearly every case the base with the beeswax attached will fit perfectly, providing you had a good impression originally. Without changing in any way, pour the palatine side of plate full of plaster of paris, building it thick and rounding over the wax and after the plaster has hardened for about twenty minutes, slightly warm the wax and remove it from between the plaster and the aluminum with an explorer. You will observe in some cases there will be space enough to admit the thickness of a sheet of ordinary base plate wax. It is now an easy matter to adapt the aluminum to the plaster by light tapping with a hammer or a horn mallet.

There is undoubtedly shinkage in other parts of the casting, but it is not detrimental to getting a good fit.

The drawing away is greatest at the heel and gradually diminishes toward the anterior ridge, producing a space which relieves the pressure over the hard center.

A cast plate is easily swaged to the plaster by light tapping on a soft stick held against the aluminum, or by holding the plaster model in the palm of hand and lightly tapping with a round nosed hammer. Do not swage up tightly before trying for fit as it is possible to swage too high, all depending on the consistency of the wax and the force used in pressing to place in the mouth. When it sticks well enough quit swaging as you can save the model and swage at any future time or can make a new one in twenty minutes. This swaging can be done after the plate is finished if necessary. It can be applied also to a swaged base, or to a vulcanite plate if considerable heat and heavy burnishing is done.

Aluminum is better than vulcanite for lower plates as it enables one to have a rigid base upon which to obtain the bite and for adjusting the teeth; they are not so easily broken and will require but little trimming if a good impression was obtained to start with.

It has been demonstrated that the common method of packing and closing flasks with moist heat, undue pressure, and too much rubber, will greatly distort the models and consequently the plates will not fit after they are vulcanized. By having aluminum bases, less rubber is required in packing; the aluminum is unyielding to the force necessary to close the flask and the plate will not be distorted.

Any dentist who can take a good impression and make a good inlay should be capable of making a cast aluminum base which will be satisfactory, provided he pays strict attention to details.—*Digest.*

